# Development of a Health Information Technology Acceptance Model Using Consumers’ Health Behavior Intention

**DOI:** 10.2196/jmir.2143

**Published:** 2012-10-01

**Authors:** Jeongeun Kim, Hyeoun-Ae Park

**Affiliations:** ^1^College of NursingSeoul National UniversitySeoulKorea, Republic Of; ^2^Research Institute of Nursing ScienceSeoul National UniversitySeoulKorea, Republic Of; ^3^Interdisciplinary Program of Medical InformaticsSeoul National UniversitySeoulKorea, Republic Of

**Keywords:** Technology Acceptance Model, health behavior, intention, consumer

## Abstract

**Background:**

For effective health promotion using health information technology (HIT), it is mandatory that health consumers have the behavioral intention to measure, store, and manage their own health data. Understanding health consumers’ intention and behavior is needed to develop and implement effective and efficient strategies.

**Objective:**

To develop and verify the extended Technology Acceptance Model (TAM) in health care by describing health consumers’ behavioral intention of using HIT.

**Methods:**

This study used a cross-sectional descriptive correlational design. We extended TAM by adding more antecedents and mediating variables to enhance the model’s explanatory power and to make it more applicable to health consumers’ behavioral intention. Additional antecedents and mediating variables were added to the hypothetical model, based on their theoretical relevance, from the Health Belief Model and theory of planned behavior, along with the TAM. We undertook structural equation analysis to examine the specific nature of the relationship involved in understanding consumers’ use of HIT. Study participants were 728 members recruited from three Internet health portals in Korea. Data were collected by a Web-based survey using a structured self-administered questionnaire.

**Results:**

The overall fitness indices for the model developed in this study indicated an acceptable fit of the model. All path coefficients were statistically significant. This study showed that perceived threat, perceived usefulness, and perceived ease of use significantly affected health consumers’ attitude and behavioral intention. Health consumers’ health status, health belief and concerns, subjective norm, HIT characteristics, and HIT self-efficacy had a strong indirect impact on attitude and behavioral intention through the mediators of perceived threat, perceived usefulness, and perceived ease of use.

**Conclusions:**

An extended TAM in the HIT arena was found to be valid to describe health consumers’ behavioral intention. We categorized the concepts in the extended TAM into 3 domains: health zone, information zone, and technology zone.

## Introduction

Since the advent of the information age, the use of diverse health information technology (HIT) has become widespread in chronic disease management, disease prevention, and health promotion. HIT is “the application of information processing involving both computer hardware and software that deals with the storage, retrieval, sharing, and use of health care information, data, and knowledge for communication and decision making” [1]. Informatics, which is another integral aspect of HIT, refers to the science of information, the practice of information processing, and the engineering of information systems. Health informatics refers to the intersection of information science, computer science, and health care, and deals with the resources, devices, and methods required for optimizing the acquisition, storage, retrieval, and use of information in health and biomedicine. Health informatics tools include not only computers but also clinical guidelines, formal medical terminologies, and information and communication systems [2]. As such, the scope of HIT is very broad and encompasses many aspects of hardware and software, including the computer, smartphone, Internet, and social network services. This advance, alongside the advancement of genomics, digitization of data collection, and storage, has inspired the concept of the health avatar [3], which is expected to cause a paradigm shift in the field of health care. The health avatar, promising active use of health and disease management technology, is enabled by life logging, self-tracking, quantified self, and more. In fact, electronics, Web service programs, and other smartphone apps that track, analyze, and provide feedback based on a person’s diet, exercise, sleeping pattern, and activity are already in public use [4].

 For effective use of collected health-related data in HIT, it is crucial that health consumers have the behavioral intention to measure, store, and manage their own data. The effort put forth by health consumers to measure, store, and manage their own data strongly determines the quality of the data. Therefore, until data collection and storage is fully automated, consumers’ behavioral intention will be the predominant deciding factor in the accuracy and usefulness of such data. Additionally, a better understanding of health consumers’ intention and behavior would aid the development and implementation of effective and efficient strategies. Thus, identifying the factors influencing health consumers’ intention and behavior to measure, store, and manage their own health-related data would enable the development of a theoretical model to successfully describe their intentions and actions. Developing a model requires determining the interrelationships among the factors of health information by integrating various behavior and information technology theories.

 First, regarding health behavior theories, health behavior includes any activity undertaken by an individual, regardless of actual or perceived health status, for the purpose of promoting, protecting, or maintaining health, whether or not such behavior is objectively effective in obtaining the intended results [5]. The best way to design programs to achieve positive changes in health behavior is to understand why people behave as they do and what might motivate them to change. Thus, theories and models of health behavior help explain consumers’ health behavior and guide the development of more effective ways to influence and change their behavior. Health behavior is, however, far too complex to be explained by a single, unified theory, and no single theory or model dominates the research or practice in health-related behavior [6]. Instead, past research devised models that drew on several theories to help understand a specific problem in a particular setting or context.

 Next, regarding the theories of information technology, the Technology Acceptance Model (TAM) is the most widely applied model to describe consumer acceptability of information technology. TAM can be adapted by applying various factors involving consumers’ behavior in the context of HIT [7,8]. TAM has been continuously expanded to TAM2, the unified theory of acceptance and use of technology, and TAM3, and each of these expansions was motivated by the need to predict the usability of new information technology and to identify and stimulate the use of the technology [9-12]. TAM considers cultural trends and social context as the main factors, and focuses on what attributes of a given technology increase consumers’ acceptance of the technology. Specifically, TAM suggests that the acceptance of a technology by consumers can be increased if efforts to improve the technology are directed by how the technology is perceived by consumers [7,8,13]. Therefore, TAM is a useful model for developing strategies to increase the acceptance of an information technology, as it provides a direct relationship between acceptance of the technology the technology’s perceived usability and ease of use. Furthermore, the applicability of TAM has been expanded by implementing theories of health behavior with the advance of HIT [14].

 The Health Belief Model (HBM) was one of the first and one of the best-known social cognition models to explain health behavior change [15]. Originally, the model was designed to predict behavioral response to the treatment received by acutely or chronically ill patients, but in more recent years the model has been used to predict more general health behaviors [6]. The HBM suggests that belief in a personal threat, together with belief in the effectiveness of the proposed behavior, predicts the likelihood of engaging in that behavior [16]. The theory of reasoned action is a model for the prediction of behavioral intention, spanning predictions of attitude and predictions of behavior. The subsequent separation of behavioral intention from behavior allows for the explanation of limiting factors on attitudinal influence. The theory of reasoned action was “born largely out of frustration with traditional attitude-behavior research, much of which found weak correlations between attitude measures and performance of volitional behavior” [17]. In psychology, the theory of planned behavior (TPB) is a theory about the link between attitude and behavior. The concept was developed to improve on the predictive power of the theory of reasoned action by including perceived behavioral control. It is one of the most predictive persuasion theories [18].

 Several key concepts, such as attitude, behavioral intention, and behavior, from these theories are the same and the paths are very similar; thus, it is possible to synthesize a model by extracting the common key concepts and building the logical paths between them. In 2008, Yun [14] built an integrated model with concepts from TAM [7,8], HBM [15], theory of reasoned action [19], and TPB [20]. The study demonstrated that consumers’ cognitive factors such as health concerns, perceived threat, and Internet self-efficacy affect their actions in seeking health information on the Internet through perceived usefulness and perceived ease of use. However, the model is limited to the pursuit of health information from the Internet, and therefore cannot comprehensively describe health consumers’ behaviors using various HITs in different environments. Therefore, the model needs to be built upon further and modified to develop TAM in the context of HIT so that it can describe consumers’ health behavior when using various HITs.

### Objectives

The objective of this study was to develop and test a model describing the behavioral intention and the health behavior of consumers of various HITs, including the Internet, smartphones, and social network services. This model, the HIT-driven extended TAM, is called the Health Information Technology Acceptance Model (HITAM).

## Methods

### Participants and Data Collection

We recruited study participants from the three largest online health portals in South Korea. They are KorMedi.com, HiDOC, and Kunkang-In (which means Healthy People, from the National Health Insurance Corporation, Republic of Korea). Members of these popular online health portals who used health information through the Internet, smartphones, or social network services were contacted for participation in the study.

 Data were collected using an online survey method developed by a private company specializing in online survey research. A survey was posted on these portals from October 21 to December 8, 2011, and in total 728 members replied.

 We sought and obtained approval from the institutional review board of the College of Nursing, Seoul National University before collecting the data.

### Measurement Tool

For this study, we developed a structured, self-administered questionnaire titled “We would like to know whether you use Smart HIT in your health management.” The questionnaire was composed of 50 items, measuring general characteristics of the research participants and variables in 10 categories. General characteristics were measured with 6 items, health belief and concerns with 5 items, subjective norm with 5 items, perceived susceptibility with 3 items, perceived seriousness with 4 items, HIT self-efficacy with 6 items, HIT reliability with 5 items, perceived ease of use with 5 items, perceived usefulness with 5 items, attitude with 3 items, and behavioral intention with 3 items. Health status was measured by asking if they had any diseases or comorbidity. (Refer to Table 4 for Cronbach alpha of each category.)

Attitude and behavioral intention are two outcome categories. Attitude measures the positive perception of and satisfaction with the use of HIT. Behavioral intention measures the intent and willingness to use HIT. Participants were asked to rate their agreement with the following 3 statements to measure attitude: (1) I am positive about using HIT to manage my health and to search for reliable health information, (2) I think it is beneficial to manage my health and search for reliable health information using HIT, and (3) I am satisfied by and large with the use of HIT to manage my health and search for reliable health information using HIT. The following 3 items measured behavioral intention: (1) I will continue to use HIT to manage my health and to search for reliable health information, (2) I will regularly use HIT to manage my health and to search for reliable health information, and (3) I will recommend use of HIT to other people to manage their health and to search for reliable health information.

We derived items measuring perceived susceptibility, perceived seriousness, perceived threat, and behavioral intention from HBM. Items measuring HIT self-efficacy, HIT reliability, perceived ease of use, perceived usefulness, attitude, and behavioral intention were derived from TAM3. Items measuring health belief and concerns, subjective norm, attitude, and behavioral intention were derived from TPB. We adapted categories of the questionnaire from Yun’s study on the development of a consumer health information-seeking behavior model from 2008 [14]. Table 1 compares Yun’s study with the present study. We made these modifications to extend and change the scope from a narrow Internet focus to broader HIT to reflect the trend of HIT adoption. Specifically, we extended the scope of the questionnaire from Internet health information use to HIT in order to better probe health consumers’ behavior.

**Table 1:** Comparison of variables in Yun [14] and the present study.

Variables in Yun’s model	Variables in proposed model	Reasons for inclusion, exclusion, or modification
Health concerns	Health belief and concerns	
Perceived susceptibility	Perceived susceptibility	
Perceived seriousness	Perceived seriousness	
Subjective health-related knowledge		Excluded because it is for the knowledge level only, which is too specific
	Subjective norm	Added to strengthen the normative beliefs such as the motivation to comply in the theory of planned behavior
Internet self-efficacy	HIT^a ^self-efficacy	Modified to adapt to HIT because it is the broader term
Perceived ease of use	Perceived ease of use	
Perceived usefulness	Perceived usefulness	
Perceived credibility	HIT reliability	Perceived credibility in Yun’s model is the same concept as the current model’s HIT reliability
Attitude	Attitude	
Behavioral intention	Behavioral intention	

^a ^Health information technology.

The measurement tool was a 7-point Likert-type scale ranging from 1 for strongly disagree to 7 for strongly agree. The questionnaire was originally created in Korean, so it was not necessary to translate it. The reliability of the original instrument was indicated by Cronbach alpha = .853. The content of the questionnaire was independently confirmed by a group of HIT experts.

### Path Diagram and Structural Equation Model

To describe health consumers’ behavioral intention toward HIT services that use computers, the Internet, and smartphones, we developed a structural equation model based on previous research and a literature review. We also analyzed other theories relevant to HITAM synthesis, such as HBM, TPB, TAM, Extended TAM (TAM2, unified theory of acceptance and use of technology, TAM3), and Consumer’s Health Information Seeking Behavior Model.

**Figure 1:**
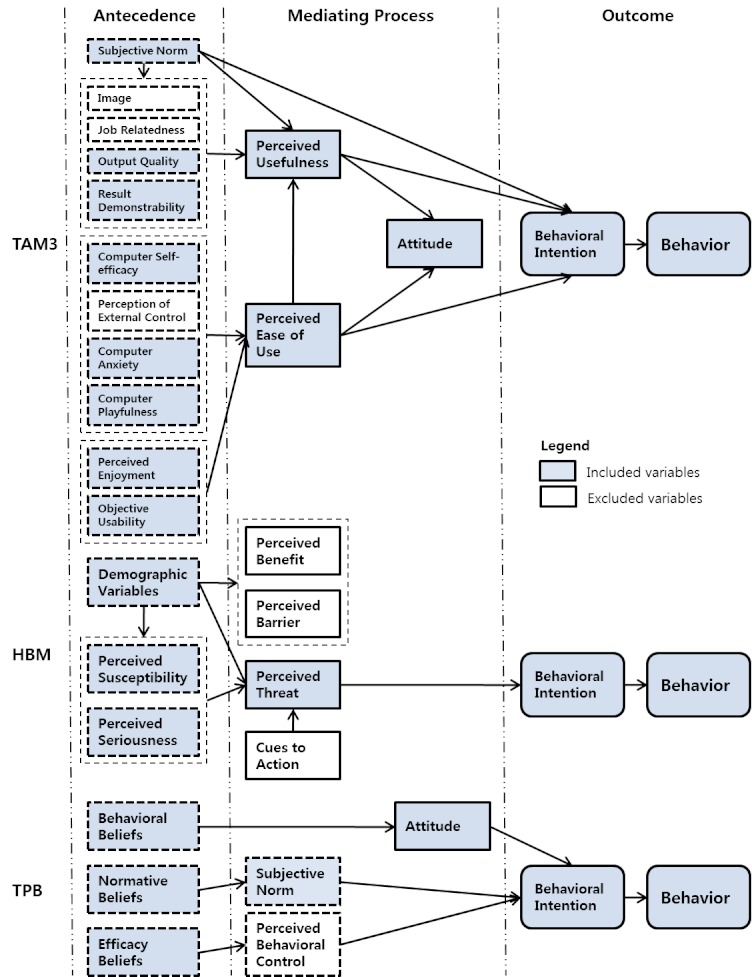
Conceptual relationships of the relevant models for development of the Health Information Technology Acceptance Model (HITAM). HBM = Health Belief Model, TAM = Technology Acceptance Model, TPB = theory of planned behavior.

Figure 1 summarizes the relationships among the models revealed by our analysis. Based on these relationships, various endogenous and exogenous variables that explain the behavioral intentions for HIT and potential variables were derived to construct a hypothetical model. We borrowed many of the concepts using their original phrasing, but we modified a few for clarification. For example, we took age and disease from the demographic category as measurement variables in the health status category that affect perceived threat. Perceived susceptibility and perceived seriousness from HBM were included as exogenous variables that affect perceived threat as well. Finally, we included behavioral belief from TPB in our health belief and concerns category.

 Extensive monitoring of behavioral change requires a long-term observational study and prohibitively large sample groups. Also, self-reported behavioral intention and users’ action itself are hard to measure. Finally, cross-sectional research methodology provides a challenge, as the correlation between behavioral intention and the actual taking of action has been shown to be weak [21,22]. Therefore, the model does not verify the previously studied relationship between health behavioral intention and health action. Rather, the influential factors and behavioral intention were the primary focus of model verification.

**Figure 2:**
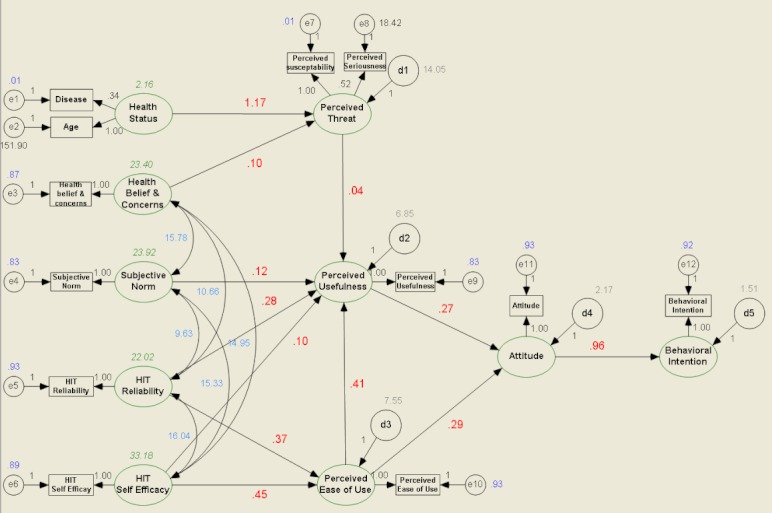
Path diagram of the Health Information Technology Acceptance Model (HITAM) Model. HIT = health information technology.

 Our structural equation model relied on findings from previous research regarding consumers’ behavioral intention to use HIT. Figure 2 shows the theoretical model for the analysis. Arrows with solid lines indicate relationships between the specified concepts. We hypothesized that consumers with worse health status and stronger health beliefs and concerns would perceive greater threat. In addition, we expected that consumers with higher subjective norm, HIT reliability, and HIT self-efficacy would perceive HIT to be more useful. We assumed that consumers with higher HIT reliability and HIT self-efficacy would perceive HIT to be easier to use. We also assumed that consumers with higher perceived threat and perceived ease of use would perceive HIT to be more useful. We hypothesized that consumers who perceive HIT to be more useful and easier to use would have a more positive attitude toward HIT. Finally, we hypothesized that consumers with a more positive attitude would show more positive behavioral intention to accept HIT.

### Statistical Analysis

Descriptive statistics of the participants’ general characteristics were analyzed with IBM SPSS version 19 (IBM Corporation, Somers, NY, USA). The structural equation model was fitted with maximum likelihood estimation routines in IBM SPSS Amos 16.

## Results

The general characteristics of the 728 research participants are shown in Table 2. About an equal number of men and women participated in the study. A large fraction of the group were in their 30s, with a majority of the group having a college education or higher. The ratio of participants with chronic illness to those without was about one.

**Table 2:** General characteristics of the participants (N = 728).

Characteristic		n	%
**Gender**			
	Male	372	51.1%
	Female	356	48.9%
**Age (years)**			
	<19	10	1.4%
	20–29	144	19.8%
	30–39	258	35.4%
	40–49	150	20.6%
	50–59	101	13.9%
	>60	65	8.9%
**Occupation**			
	Clerical	223	30.6%
	Professional	113	15.5%
	Homemaker	86	11.8%
	Student	75	10.3%
	Self-employed	58	8.0%
	Manufacturing	33	4.5%
	Government official	24	3.3%
	Other	116	15.9%
**Education**			
	< Middle school	10	1.4%
	High school	177	24.3%
	College	135	18.5%
	University	299	41.1%
	> Graduate school	107	14.7%
**Chronic diseases**			
	Yes	361	49.6%
	No	367	50.4%
**Monthly income (US $)**			
	>1000	37	(5.1)
	1001–2000	107	(14.7)
	2001–3000	186	(25.5)
	3001–4000	159	(21.8)
	4001–5000	118	(16.2)
	<5000	121	(16.6)

The descriptive statistics of the variable scores and the reliability coefficients of the measuring tool in each category are provided in Table 3.

**Table 3:** Descriptive statistics of the latent variables and the reliability coefficients (N = 728 for all variables).

Variable	Minimum	Maximum	Mean	SD	Kurtosis		Skewness		Cronbach alpha	No. of items
Health belief and concerns	5	35	27.04	4.930	–1.028	0.091	2.405	0.181	0.867	5
Subjective norm	5	35	23.68	4.978	–0.318	0.091	0.289	0.181	0.826	5
Perceived susceptibility	3	21	12.94	4.157	–0.291	0.091	–0.448	0.181	0.751	3
Perceived seriousness	4	28	20.91	4.801	–0.844	0.091	0.811	0.181	0.907	4
HIT^a ^self-efficacy	6	42	31.25	5.841	–0.412	0.091	0.693	0.181	0.888	6
HIT reliability	5	35	25.55	4.795	–0.485	0.091	0.521	0.181	0.934	5
Perceived ease of use	5	35	27.74	4.847	–0.716	0.091	0.734	0.181	0.925	5
Perceived usefulness	5	35	25.99	4.710	–0.466	0.091	0.364	0.181	0.826	5
Attitude	3	21	16.76	3.024	–0.872	0.091	1.088	0.181	0.93	3
Behavioral intention	3	21	17.07	3.146	–0.921	0.091	0.956	0.181	0.919	3

^a ^Health information technology.

 We found that each measured variable satisfied the assumption of the univariate normality. Table 4 presents correlation coefficients for the measured variables. The correlations between the measured variables ranged from .001 to .817.

**Table 4:** Correlation coefficients between measured variables.

	Age	Diseases	Health belief and concerns	Subjective norm	Perceived susceptibility	Perceived seriousness	HIT^a^ self- efficacy	HIT reliability	Perceived ease of use	Perceived usefulness	Attitude	Behavioral intention
Age	1											
Diseases	–.117**	1										
Health belief and concerns	.144**	–.023	1									
Subjective norm	.049	.025	.644**	1								
Perceived susceptibility	.032	–.411**	.119**	.151**	1							
Perceived seriousness	.057	–.156**	.340**	.207**	.447**	1						
HIT self-efficacy	–.053	.056	.517**	.529**	.155**	.288**	1					
HIT reliability	.048	.031	.449**	.405**	.143**	.316**	.574**	1				
Perceived ease of use	.002	.024	.488**	.403**	.139**	.350**	.733**	.655**	1			
Perceived usefulness	–.009	.026	.476**	.479**	.175**	.370**	.664**	.672**	.740**	1		
Attitude	.097**	.001	.520**	.387**	.126**	.358**	.640**	.694**	.740**	.737**	1	
Behavioral intention	.145**	.014	.496**	.361**	.110**	.350**	.635**	.604**	.705**	.686**	.817**	1

^a ^Health information technology.

*P < .05, **P < .01.

Figure 2 presents the path diagram of the fitted model, and Table 5 shows unstandardized and standardized estimates of the model. This model exhibited an excellent fit to the data (χ^2^_46 _= 375.6, *P *< .001, goodness of fit index = .923, root mean square error of approximation = .099). The model accounts for 83% of the variance in behavioral intention, 73% of the variance in attitude, 18% of the variance in perceived threat, 67% of the variance in perceived ease of use, and 68% of the variance in perceived usefulness. The path from perceived threat to behavioral intention was proposed in the hypothetical model. However, we removed this path in the final model because it was not significant.

**Table 5:** Standardized estimates of the Health Information Technology Acceptance Model (HITAM).

Endogenous variable	Exogenous variable	Regression weights (SE)	Standardized estimate (beta)	CR^a^ (*t *value)	*P *value	SMC^b^
Perceived threat	Health status	1.167 (0.379)	.413	3.078	.002	.184
	Health belief and concerns	0.100 (0.029)	.117	3.415	<.001	
Perceived ease of use	HIT^c ^reliability	0.372 (0.030)	.367	12.305	<.001	.665
	HIT self-efficacy	0.446 (0.024)	.542	18.275	<.001	
Perceived usefulness	Subjective norm	0.118 (0.026)	.126	4.475	<.001	.677
	HIT reliability	0.276 (0.033)	.281	8.381	<.001	
	Perceived ease of use	0.409 (0.041)	.422	9.996	<.001	
	Perceived threat	0.045 (0.025)	.040	1.777	.08	
	HIT self-efficacy	0.102 (0.032)	.127	3.218	.001	
Attitude	Perceived usefulness	0.268 (0.024)	.432	11.363	<.001	.734
	Perceived ease of use	0.288 (0.023)	.479	12.588	<.001	
Behavioral intention	Attitude	0.955 (0.025)	.912	38.669	<.001	.831

^a ^Critical ratio.

^b ^Squared multiple correlation.

^c ^Health information technology.

Table 6 displays standardized measures of direct, indirect, and total effects of exogenous variables on endogenous variables in the model. All direct, indirect, and total effects were statistically significant except for the direct effect of perceived threat on perceived usefulness, the indirect effect of perceived threat on attitude, and the indirect effect of perceived threat on behavioral intention.

**Table 6:** Effects of exogenous variables on endogenous variables in the Health Information Technology Acceptance Model (HITAM).

Endogenous variable	Exogenous variable	Standardized direct effect	*P *value	Standardized indirect effect	*P *value	Standardized total effect	*P *value
Perceived threat	Health status	.413	.01	0		.413	.01
	Health belief and concerns	.117	.006	0		.117	.006
Perceived ease of use	HIT^a ^self-efficacy	.542	.007	0		.542	.007
	HIT reliability	.367	.01	0		.367	.01
Perceived usefulness	HIT self-efficacy	.127	.009	.228	.02	.356	.009
	HIT reliability	.281	.009	.155	.009	.436	.008
	Subjective norm	.126	.01	0		.126	.01
	Perceived threat	.040	.06	0		.040	.06
	Perceive ease of use	.422	.008	0		.422	.008
	Health belief and concerns			.005	.04	.005	.04
	Health status			.017	.05	.017	.05
Attitude	Perceived ease of use	.479	.02	.182	.005	.661	.02
	Perceived usefulness	.432	.006	0		.432	.006
	HIT reliability			.364	.03	.364	.03
	HIT self-efficacy			.413	.01	.413	.01
	Subjective norm			.054	.006	.054	.006
	Health belief and concerns			.002	.03	.002	.03
	Health status			.007	.046	.007	.046
	Perceived threat			.017	.05	.017	.05
Behavioral intention	Attitude	.912	.01	0		.912	.01
	HIT reliability			.332	.03	.332	.03
	HIT self-efficacy			.377	.01	.377	.01
	Subjective norm			.049	.006	.049	.006
	Health belief and concerns			.002	.04	.002	.04
	Health status			.007	.04	.007	.04
	Perceived threat			.016	.05	.016	.05
	Perceived ease of use			.603	.01	.603	.01
	Perceived usefulness			.394	.005	.394	.005

^a ^Health information technology.

## Discussion

We categorized the influential factors affecting the behavioral intention to measure, store, and manage health-related data into three domains called the health zone, information zone, and technology zone. In each zone, the factors follow different mediating processes that lead to behavioral intention in health customers. In the first domain, the health zone, there is a cascade of effects starting from health status (age, disease, etc), to perceived threat, to perceived usefulness, to attitude and, finally, to behavioral intention. This further verifies the description of behavioral intention in the HBM, where an intermediate variable, attitude, leads to behavioral intention [23,24]. Additionally, we showed that health belief and concerns have an indirect effect on the behavioral intention to use HIT. This suggests that psychological incentives such as health belief and concerns may motivate people to take action toward their health management. For example, outcome valuation has a nonnegligible effect on the health belief and concerns, triggering behavioral intention in health consumers.

 Consumers’ perceived threat, which is measured by perceived susceptibility and perceived seriousness, had a somewhat smaller impact on perceived usefulness than in previous studies. However, perceived threat had a statistically significant indirect effect on attitude through perceived usefulness, subsequently increasing the behavioral intention to use HIT. This indirect relationship is exemplified by the tendency of individuals to actively use HIT when they perceive a potential threat to their health. This result is consistent with the model reported by Yun [14], where perceived threat leads to information-seeking behavior. One possible explanation for the relatively reduced impact of perceived threat on perceived usefulness found in this study may be that some research participants considered the issue of susceptibility to and seriousness of disease not to apply to themselves, owing to their good health. Another explanation may be that perceived susceptibility and perceived seriousness are concerned with disease, but perceived usefulness is concerned with the usefulness of HIT in health promotion.

 In the second domain, the information zone, the factors have a similar cascade effect to that in the health zone. Perceived usefulness is significantly sensitive to subjective norms, such as social pressure or community competition, resulting in consumers forming positive attitude. Such a formed attitude has a consequence on behavioral intention. According to the Ajzen’ TPB [18], behavioral intention is a direct determinant of behaviors, arguing that attitude, subjective norm, and perceived behavioral control of the action are the most powerful predicting factors for behavioral intention. Therefore, the result suggests that when using HIT to induce health behaviors in consumers, using a social network service or network to form an environment for users to compete with others online can increase efficiency.

 The last domain, the technology zone, has factors with the following characteristics. HIT use forms specific HIT reliability, such as output quality and result demonstrability, and these affect perceived usefulness. More interestingly, HIT reliability also affects perceived ease of use, also affecting perceived usefulness. Similarly, HIT self-efficacy, such as HIT anxiety, playfulness, perceived enjoyment, and objective usability, also affect perceived usefulness and perceived ease of use. These two factors affect attitude and, finally, form behavioral intention. Our study revealed that HIT self-efficacy is a significant factor influencing HIT use. This suggests that consumers enjoying the use of HIT and gaining confidence in their ability to use HIT develop increased tendencies to use HIT. Specifically, the greater the self-efficacy, the greater the perceived ease of use, which is consistent with the finding reported by Yun [14].

 Ajzen developed the TPB model that explains various human actions by integrating behavioral belief, normative belief, efficacy belief, attitude, subjective norm, and perceived behavioral control [18]. In this study, we demonstrated that behavioral belief, normative belief, and efficacy belief all serve as antecedents to a mediating process that results in behavioral intention. The similarity between these two studies suggests that the TPB is applicable to further research in HIT.

 The key factors identified within the three zones—health status and health belief and concerns in the health zone; subjective norms and HIT reliability in the information zone; and HIT self-efficacy in the technology zone—are the predicting factors that form the HITAM with varying ranges of significance and directional relationships. By identifying the core factors that have the largest impact, HITAM is a succinct and powerful model that reevaluates and reorganizes previous findings in the field.

 The survey revealed that, although TAM has expanded its utility into various areas and has been successfully implemented, its implementation in the HIT arena has been limited. Furthermore, even in the rapid advancement of information technology and its subsequent impact on health management, a model that captures and predicts various aspects of consumer acceptance is lacking. To address this gap, our research has tried to provide a robust foundation for future HIT research. Yun’s similar effort in integrating health information-seeking activities with TAM was limited to the use of the Internet and did not capture the real-life application of HIT. Therefore, by considering the latest developments in HIT, now used more ubiquitously, the model in this work possesses increased real-life applicability and predictive capability.

**Figure 3:**
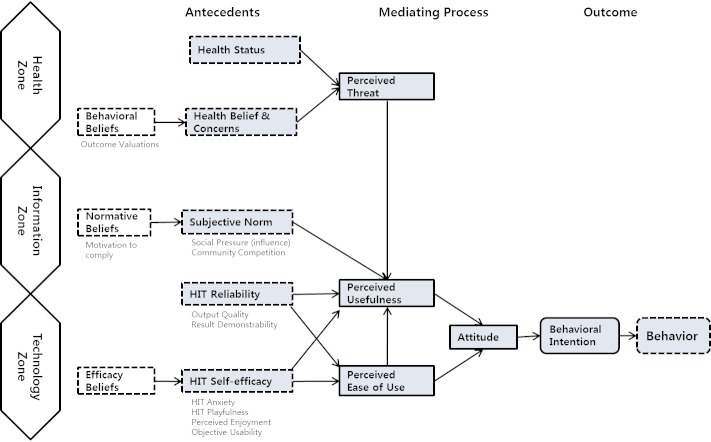
The Health Information Technology Acceptance Model (HITAM). HIT = health information technology.

Figure 3 shows the overall structure of HITAM, which describes health consumers’ attitude and behavioral intention when encountering HIT. We discuss five distinct factors in this work: health status, health belief and concerns, subjective norm, HIT reliability and HIT self-efficacy. The first two factors lead to a mediating process of perceived threat toward consumers’ health. When consumers consider the possibility of deteriorating health, they may seek the use of HIT to better manage their health. This leads to the consumer determining the perceived usefulness of HIT, and in the case of positive interaction, it results in positive attitude, which ultimately leads to their behavioral intention. The third factor, subjective norm, concerns social pressure or competition within the community. When health consumers discover a given HIT over social network services or other means of online interaction, they are likely to perceive the HIT as useful. This encounter, depending on their experience, leads to positive attitude, and in turn leads to their behavioral intention. The fourth antecedent, HIT reliability, is unique in that it lies in both the information zone and the technology zone. HIT reliability includes quality of output and demonstrability of result, which suggests that it can come from either a direct experience with the technology or an indirect experience, gained through information gathered through other consumers. On the other hand, it also leads to perceived ease of use. This particular mediating process can lead immediately through perceived usefulness, because a user who finds a given information technology easy to use will generally perceive it to be useful as well. The final antecedent, HIT self-efficacy, follows a similar path toward perceived usefulness and perceived ease of use, which can arise from health consumers’ confidence in using a given technology. The mediating processes lead to positive attitude, which in the HITAM is a crucial prerequisite to forming behavioral intentions in health consumers.

 Using this model, many aspects of health behaviors using HIT can be explained. Thus, in the wake of the exponentially increasing presence of HIT such as the Internet and smartphone apps, the HITAM provides a valuable model of how different interactions with HIT form behavioral intention in health consumers.

## Abbreviations

HBM: Health Belief Model
HIT: health information technology
HITAM: Health Information Technology Acceptance Model
TAM: Technology Acceptance Model
TPB: theory of planned behavior
